# Chondroid Chordoma and Nasal Adenocarcinoma: An Exceptional Association

**DOI:** 10.1155/2012/861217

**Published:** 2012-09-17

**Authors:** Patrice Gallet, Nathalie Marcon, Thomas Georgel, Jean-Marie Vignaud, Cécile Parietti-Winkler, Roger Jankowski

**Affiliations:** ^1^Department of ENT and Cervico-Facial Surgery, University Hospital, University of Lorraine, 54003 Nancy Cedex, France; ^2^Department of Pathology, University Hospital, University of Lorraine, 54003 Nancy Cedex, France

## Abstract

Collision tumors are exceptional, associating two independent tumoral contingents. We report a case of an association of two rare tumors: sinonasal adenocarcinoma and chondroid chordoma. Initially, only adenocarcinoma was diagnosed. The treatment consisted of endoscopic endonasal surgery followed by conventional radiotherapy. After 18 months, a local recurrence was diagnosed after a facial trauma, but the true histology was difficult to assess. The tumor was dual, associating adenocarcinoma and chondroid chordoma, with atypical localization in the ethmoid. Further evolution was particularly aggressive. We discuss the key points of this observation.

## 1. Introduction

Sinonasal adenocarcinoma and chondroid chordoma are both rare tumors.

The incidence of sinonasal adenocarcinoma is less than 1 per 100,000 per year. The peak incidence is in the 5th to the 7th decades [1]. Woodworkers are particularly exposed. Occupational exposure to nickel or leather dust has also been reported as favoring adenocarcinoma. In woodworkers, adenocarcinomas are usually of intestinal type (ITAC) [2].

Chordomas account for 2 to 4% of primary bone tumors, being the fourth most common pathology among primary bone cancers. The annual incidence is estimated to 1 per 1000,000, with a peak incidence in the 4th to 7th decade [3].

We report and discuss here the first case of association of these two rare tumors.

## 2. Case Report

A 63-year-old man presented with a history of nasal obstruction and recurrent epistaxis for two months. He was woodworker for more than 40 years (flooring installer). He had no smell anymore for 20 years, which was thought to be due to the intensive use of varnish and glue. He also described a discrete right ptosis for several months. There were no additional symptoms. The patient had a history of hypertension, hypercholesterolemia, and septoplasty 30 years prior, for nasal obstruction.

**Figure 1:**
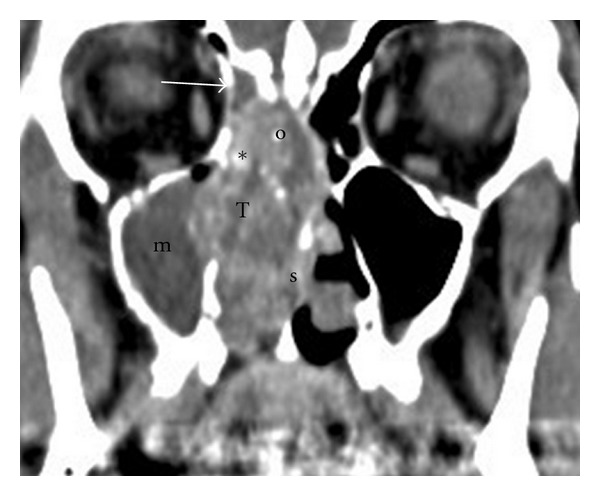
Initial lesion, CT scan: the tumor (T) is centered on the olfactory cleft (o), pushing the septum (s). The invasion of the middle turbinate and the presence of intratumoral calcifications (∗) could indicate the presence of chordoma. Ethmoidal attic (*→*) and maxillary sinus (m) are retentional.

**Figure 2:**
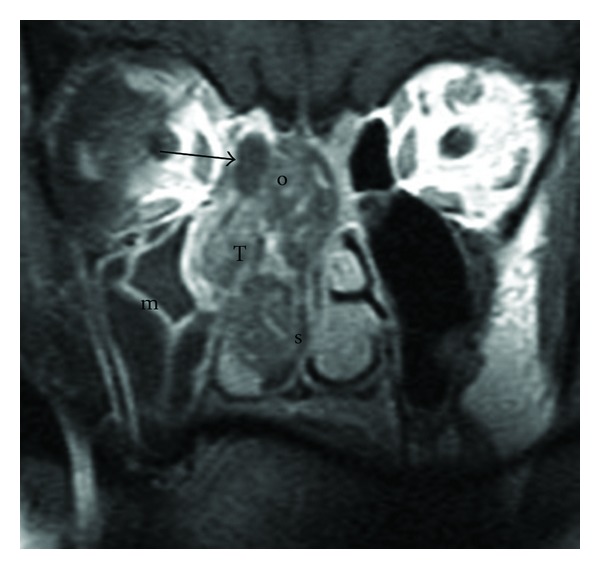
Initial lesion, MRI: MRI confirms CT scan images (T: Tumor, o: olfactory cleft, *→*: ethmoidal attic, and m: maxillary sinus). There is no typical signal of chordoma (T1 hyposignal and T2 very intense hypersignal).

An initial examination found a polyp masking the olfactory cleft with many mucopurulent secretions. An antibiotherapy treatment (amoxicillin + clavulanic acid) and steroids were prescribed and a CT scan was performed. The CT scan highlighted a tissular process invading the posterior part of the ethmoid, the olfactory cleft, and the nasal septum, with swelling in the maxillary sinus. There was no evidence for skull base invasion or intracranial extension. The tumor was partially ossified (Figure 1). There was no regional evolution or metastasis. After two weeks of antibiotics, the examination could find this tumor located in the olfactory cleft, which was biopsied under local anesthesia. The histopathologist reported a well-differentiated infiltrating intestinal-type adenocarcinoma. MRI confirmed the CT scan findings (Figure 2).

**Figure 3:**
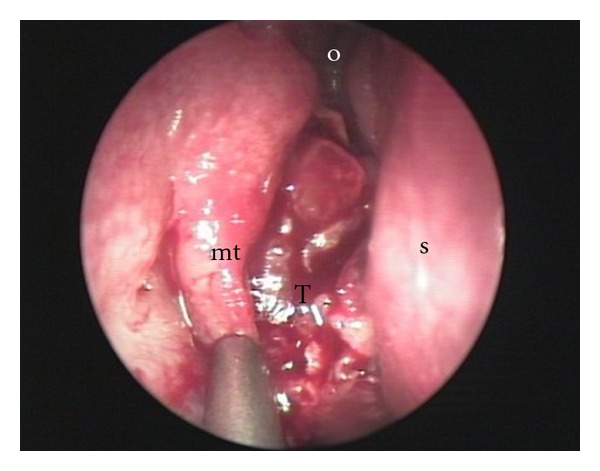
Initial lesion, endoscopic view: the tumor (T) is centered on the olfactory cleft (o), pushing the septum (s) medially and the middle turbinate (mt) laterally.

The patient underwent endoscopic surgery (Figure 3). After an initial debulking, the tumor appeared pedunculated on the medial and superior part of the middle turbinate. The exeresis began with the resection of the bony orbital wall, continued with the dissection of the ethmoid roof, then the exenteration of the olfactory cleft, with resection of the posterior part of the septum. The tumor was invading the posterior ethmoid and the posterosuperior part of the nasal septum. Laterally, there was a lysis of the orbital lamina in front of Haller cells. The orbital content was respected by the tumor. Posteriorly, the tumor reached the anterior sphenoid wall and the sphenopalatine foramen. The sphenoid sinus and the maxillary sinus were full of retention, though their mucosae seemed safe. The histopathological examination confirmed the surgical findings and the exeresis was complete. After multidisciplinary discussion, 56 grays were delivered to the CTV (6 MeV photons), by conformational fractionated radiotherapy in 28 fractions.

Patient followup was marked by the persistence of nasal obstruction because of crusts, requiring repeated and frequent local care, but without sign of recurrence.

Eighteen months afterward, the patient presented with an increasing trismus, four weeks after a local trauma. It was associated with hypoesthesia and pain in the infra-orbital area. The CT scan highlighted a zygoma fracture. There was also a partial lysis from the posterolateral and the lateral wall of the maxillary sinus. This was considered to be highly suspicious, but as the endoscopic control found no evidence for recurrence, the first diagnosis retained was osteoradionecrosis. The patient was treated with pristinamycin, metronidazole, and pentoxifylline, with a control by CT scan and MRI at one month.

The pejorative clinical and radiological progression—with palsy of the right oculomotor nerve—led to the realization of biopsies.

Histopathological examination revealed a cartilaginous tumor with immature chondrocytes. Their nuclei were irregular, with densified chromatin and frequent nucleoli. The stroma was myxoid without lobular organization, with inflammatory infiltration and foci of necrosis. The diagnosis retained was grade II chondrosarcoma. A second biopsy led to the same diagnosis.

After complete evaluation (CT scan, MRI, and PET-CT), the tumor was found to be centered on the maxillary sinus, invading its posterolateral and anterolateral walls, the infra-temporal fossa, and the fat space of the cheek, with bilateral node invasion, but without distant metastasis. Multidisciplinary discussions led to the proposition of a right maxillectomy with bilateral lymph node dissection.

The tumor was approached through a Weber-Ferguson incision. The exeresis was extended laterally to the zygoma, upper to the superior part of the malar and periorbital soft tissues, medially to the right nasal bone, inferiorly to the homolateral hard palate, and posteriorly to the infratemporal fossa.

The final histologic examination excluded the diagnosis of chondrosarcoma and revealed a dual tumor. There were foci of the initial adenocarcinoma, but the main contingent was composed of a chondroid chordoma. The chondroid component, mimicking a grade II chondrosarcoma was intricate with an epithelial component. Cells were organized in a trabecular, cribriform, or acinous mode, with a high nucleocytoplasmic ratio, hyperchromic nuclei, with thin chromatin and large nucleoli. Atypical mitoses were frequent. These areas of epithelial differentiation were sometimes included in a hyaline stroma. Some physaliphorous cells could be observed. Diagnosis was confirmed by immunohistochemical staining. Cells strongly expressed S-100 protein and were positive for cytokeratin AE1-AE3, EMA (epithelial membrane antigen), and at a lower level for cytokeratin 19; margins were not clear. Lymph nodes were massively invaded by adenocarcinoma with capsular effraction. One subcutaneous lymph node was invaded by chondroid chordoma.

**Figure 4:**
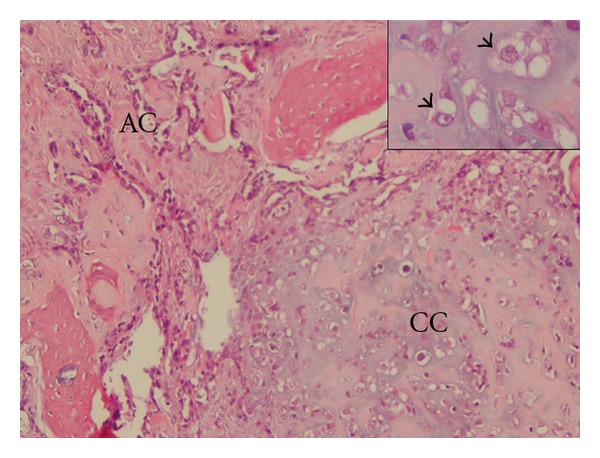
Anatomo-pathologic examination of the primary lesion (ethmoidal samples): the two tumoral contingents are present at the outset: adenocarcinoma (AC) is in contact with chondroid chordoma (CC). Physaliphorous cells are visible (→). Their nuclei are eccentric, with prominent nucleoli and cytoplasmic vacuoles of mucoid material.

 As the presentation of this case was unusual, the diagnosis of chordoma was confirmed by a second laboratory and the histological slides of the first tumor were reviewed. This lead to the discovery of a small contingent of chondroid chordoma, lost in the adenocarcinoma, in ethmoidal samples. The collusion of the two tumors was present at the outset (Figure 4). However, the sphenoidal samples, the orbital wall, the septal part of the olfactory cleft, the ethmoidal roof, and the cribriform plate were found to be free of the chordoma.

After multidisciplinary discussion, as no additional radiotherapy could be performed, targeted therapy was proposed with Imatinib.

However, the situation worsened with a rapid course progression, no objective response to targeted therapy, local evolution, and pulmonary metastasis.

The patient died shortly thereafter, two years after initial diagnosis.

## 3. Discussion

The association of chondroid chordoma and nasal adenocarcinoma has never been described before. This case raises the question of a possible link between both tumors.

The physiopathology of chordoma remains unclear; chordoma derive from the primitive notochord. The initial lesions might be benign chordal ectopias called ecchordosis physaliphora, which are encountered in asymptomatic adults with an incidence of 1% [3].

However, no precise responsible locus could be found in the different genomic studies, despite several different chromosomic abnormalities identified [3]. Likewise, little is known about ITAC's oncogenesis. Exposure to hardwood, leather dust, or other unknown agents seems to induce metaplasia in the respiratory mucosa (goblet cell hyperplasia, cuboidal metaplasia [4]). The activation of CDX-2 may enable the acquisition of a full intestinal phenotype [2]. Then, additional genetic events like the p53 mutation [5] may be required for the development of invasive adenocarcinoma. Mutation of p53, which is for both tumors correlated with decreased survival [3, 5], is to our knowledge the only common cytogenetic abnormality between the two tumors, so that this association is probably fortuitous.

Nevertheless, the collision of the two tumors seems to have influenced their evolution. In a general manner, adenocarcinomas do not have a very aggressive behavior, which is reflected in their low metastatic potential (1-2%) [1], and their development that respects the surrounding structures. Adenocarcinomas usually originate in the olfactory cleft and tumoral growth is highly limited by the successive encountered barriers [6]. The unfavorable prognosis of these tumors (overall mortality of 53% [1]) is mainly due to late diagnosis, as symptoms are subtle and aspecific (anosmia, nasal obstruction, epistaxis, etc) [7]. Chordomas also have a low course evolution [3]. They usually tend to displace surrounding soft tissues more than invading them. Tumors are able to display proteolytic activity, and may be widely invasive in bone tissue, but generally metastases occur late in the disease course, usually years after the initial diagnosis [3]. Skull base lesions are less likely to metastasize than sacrococcygeal and vertebral chordomas. The incidence of metastases in chordomas, regardless of their location and their initial size, is estimated to be between 3 and 48% [3]. Here, the evolution was significantly more aggressive than expected for these tumors, with local recurrence, lymph node invasion for both tumors, and pulmonary metastasis for adenocarcinoma; though there was no histological evidence of interaction between the two lesions. Despite close proximity, both tumors kept well organized like independent tissues. We postulate that it is due to the well-differentiated organization of the two contingents, so that the organization was more influenced by cell phenotypes than by stromal signals [8]. Though, the presence of another tumoral contingent might also have had a stimulating effect on cell proliferation [8]. Tumoral expansion may also have been favored by the fracture: stromal wound healing stimulates cancer growth and spread [9]. Inflammatory responses are decisive at different stages of tumor development and inflammation also affects immunity [9]. In this case, the trauma obviously played a stimulating role as tumoral expansion clearly accelerated after the fracture.

Another interesting aspect of this case is the initial localization of the chordoma in the ethmoid. Chondroid chordoma are thought to derive from the notochord. The notochord is a transient structure of embryogenesis, which arises as a pouch from the mesoderm during gastrulation. It defines the primitive axis of the embryo, conditions its elongation, and the formation of the neural tube. In higher vertebrates, it regresses completely throughout the column except in the nucleus pulposus. This process explains why chordomas are located along the axial skeleton (from the sacrum to the sphenoidal clivus) [10]. Extra axial localizations remain possible but exceptional, so that the presentation of this case was very surprising. The chordoma was indeed exclusively localized in the ethmoid, without involvment of the sphenoid sinus, the cribriform plate, the ethmoidal roof, or the orbital wall (in a retrospective analysis of initial pathological samples). The ethmoid has a very different embryological origin as it derives from the nasal capsule. This capsule is normally separated from the notochord by the trabeculae, derived from neural crest cells, which will later become the sphenoid bone. The isolated impairment of the ethmoid bone cannot be explained embryologically. Even more surprising, the recurrence arose essentially from the maxillary bone in its lateral and anterior part.

The localization of the adenocarcinoma was more typical. In a recent report, we pointed out that adenocarcinomas seem to arise from the olfactory cleft [6], so that the clinical and radiological screening for adenocarcinoma in exposed workers should be focused on this localization. An opacity located in the olfactory cleft on CT-scan is always suspected, and the radiologist should be warned that it must not be taken for polyposis [11]. In our case, the tumor was initially clearly centered on the olfactory cleft. Ethmoidal cell invasion usually occurs relatively late in the development of adenocarcinoma, as these cells are protected by the middle turbinate which is pushed laterally. The posterior ethmoidal extension may therefore have been favored by the presence of chondroid chordoma, which has a greater capacity for bone invasion.

This case illustrates the difficulty in diagnosing chondroid chordoma. The similarities with chondrosarcoma are so important that the existence of chondroid chordoma has long been questioned [3, 12]. The classic histopathological characteristic of chordoma is the presence of physaliphorous cells [13]. These cells contain an intracytoplasmic accumulation of mucopolysaccharides. Their nuclei are vacuolated, eccentric, with prominent nucleoli. Classical chordoma is organized in a lobular pattern, with cords of epithelioid cells within a mucomyxoid matrix. Cells rarely present atypia. In chondroid chordoma, within features of classical chordoma, areas of chondroid differentiation are observed, mimicking a low-grade chondrosarcoma. Differential diagnosis can only be assessed by immunochemistry. Chordomas are positive for EMA (epithelial marker antigen), cytokeratins (CK19), and S-100 protein staining [14]. Chondroid areas are positive for vimentin staining [14]. Chondrosarcomas do not express epithelial markers, and are positive only for vimentine staining. Finally, D2-40 staining has proved to be effective in distinguishing true chordoid tumors from chondrosarcoma, but is not currently used [15]. In our case, the localization and the biopsies were strongly in favor of chondrosarcoma rather than of chordoma. Our first hypothesis was radio-induced chondrosarcoma, even if the delay was short, as trauma might have accelerated the progression, but it was subsequently discovered that small foci of chondroid chordomas were present at the outset, and the immunochemistry contradicted this hypothesis. However, this highlights the importance of large-volume biopsies, representative of the tumor, to be ideally carried out under general anesthesia.

Therapeutic strategy is based on aggressive but safe surgery for both tumours [1, 3].

Since our understanding that adenocarcinomas arise from the olfactory cleft, we have developed an endoscopic approach instead of lateral rhinotomy [6, 16]. This approach, derived from the nasalisation technique, usually enables the discovery of the pedicle of the tumor and a safe resection. Here, recurrence might have been favored by the association with chondroid chordoma, as most chordomas recur after surgical resection. But one cannot rule out the hypothesis of an intraoperative tumoral dissemination during the surgical act. Endoscopic resection transgresses one fundamental oncological concept, as it requires piecemeal removal, which might favor dissemination. But in fact, in sinonasal surgery, true “en bloc” resection is rarely possible, even with open approaches, the exeresis is not easier and may also cause intraoperative dissemination [16]. There is no study showing that piecemeal removal increases local recurrences [17]. In the literature, as in our experience, an endoscopic approach seems promising [18–20], but its precise indications remain to be precisely defined. 

Adenocarcinomas and chordomas have a poor radiosensitivity [1, 3]; this case is no exception to the rule. As chordomas have an intermediate proliferative index and probably an intermediate *α*/*β* ratio for linear quadratic modeling, conventional radiotherapy has often been associated with particle beam radiotherapies or radiosurgery, which enables higher doses.

For both tumors, conventional chemotherapy is usually only used in palliative intention [1, 3]. For chordomas, protocols are based on chemotherapies intended for sarcomas. The presence of the PDGF receptor on chordomas cell membranes was demonstrated by Casali et al. and imatinib was proposed and showed some evidence for clinical benefit [21]. For adenocarcinomas, chemotherapy has been used preoperatively by Brasnu et al.with good results for some patients [22]. 5-Fu has been applied locally by Knegt et al. with excellent local control (87% at 5 years) [23]. Finally, the EGF R inhibitor may theoretically be an interesting treatment as adenocarcinomas exhibit these receptors [1]. 

## 4. Conclusion

This is the first case of the association of chondroid chordoma and adenocarcinoma of the olfactory cleft. Our report features a particularly aggressive example of these two rare tumors, with an exceptional localization of the chordoma. It illustrates the difficulty in diagnosing chondroid chordoma, questions the endonasal endoscopic approach, and confirms the poor radiosensitivity of both tumors.
